# Guided by Microbes: Applying Community Coalescence Principles for Predictive Microbiome Engineering

**DOI:** 10.1128/mSystems.00538-21

**Published:** 2021-08-17

**Authors:** Jennifer D. Rocca, Mario E. Muscarella, Ariane L. Peralta, Dandan Izabel-Shen, Marie Simonin

**Affiliations:** a Department of Plant and Microbial Biology, North Carolina State University, Raleigh, North Carolina, USA; b Institute of Arctic Biology, Department of Biology and Wildlife, University of Alaska Fairbanks, Fairbanks, Alaska, USA; c Department of Biology, East Carolina University, Greenville, North Carolina, USA; d Department of Ecology, Environment, and Plant Sciences, Stockholm University, Stockholm, Sweden; e University of Angers, Institut Agro, INRAE, IRHS, SFR QUASAV, Angers, France; Michigan State University; Yale University

**Keywords:** biostimulants, community coalescence, community inoculants, managed ecosystems, microbiome engineering

## Abstract

Every seed germinating in soils, wastewater treatment, and stream confluence exemplify microbial community coalescence—the blending of previously isolated communities. Here, we present theoretical and experimental knowledge on how separated microbial communities mix, with particular focus on managed ecosystems. We adopt the community coalescence framework, which integrates metacommunity theory and meta-ecosystem dynamics, and highlight the prevalence of these coalescence events within microbial systems. Specifically, we (i) describe fundamental types of community coalescences using naturally occurring and managed examples, (ii) offer ways forward to leverage community coalescence in managed systems, and (iii) emphasize the importance of microbial ecological theory to achieving desired coalescence outcomes. Further, considering the massive dispersal events of microbiomes and their coalescences is pivotal to better predict microbial community dynamics and responses to disturbances. We conclude our piece by highlighting some challenges and unanswered question yet to be tackled.

## PERSPECTIVE

## COMMUNITY COALESCENCE: DEFINITION, OCCURRENCES, AND KNOWLEDGE SYNTHESIS FROM CASE-STUDIES

Community coalescence—dispersal en masse—is a pervasive process in the microbial realm (1–4). For example, leaf and soil microbiomes make contact and integrate during litterfall (5). Blending separated communities often occurs in tandem with their respective environments, characteristic of stream confluences (2). Community coalescence can be ephemeral (e.g., monsoonal erosion) or perpetual (e.g., tidal ebb/flow), with restricted spatial distribution (e.g., bird fecal deposits) or be widespread (soil blowing across the landscape). These naturally occurring coalescences may be important components of ecosystem function but are also key features of managed systems where the coalescences are intentionally implemented or controlled (1, 6).

**FIG 1:**
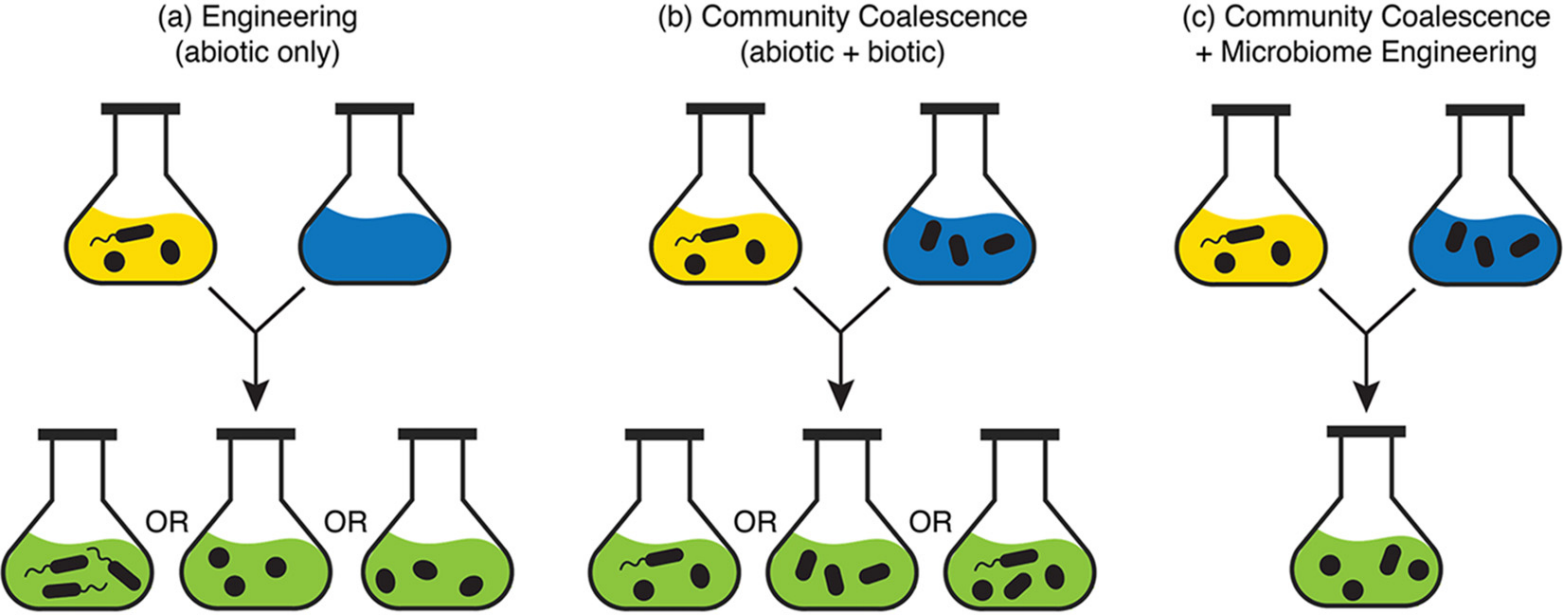
Conceptual representation of intentional microbiome management. The three panels depict how (a) engineering with regard to manipulating abiotic conditions only or (b) mixing two previously isolated communities and associated environments can result in different community outcomes; in contrast (c), combining approaches of intentional microbiome engineering of specific synthetic communities and considering/addressing how environmental conditions affect resultant community assembly refines the predictability of microbiome management.

Microbial engineering historically focused on isolating and imposing directed artificial selection, or selective enrichment, on one or a few microorganisms, to optimize the strain(s) for a particular function or set of functions. This approach largely overlooks the biotic interactions with the receiving community for the success/failure of these strains (7–9) (Fig. 1a). Embracing a top-down approach, Swenson et al. introduced the concept of “ecosystem selection” (10), wherein whole ecosystems, comprising many species and their interactions, are targeted and tested for optimizing engineered systems, without the colossal task of examining every species or all possible interactions. A recent study demonstrates that applying repeated perturbations on microbial communities as an artificial selection strategy results in microbial consortia with high functionality and invasion resistance (11). As such, using wholescale mixing of isolated communities in designing microbial consortia (Fig. 1b) should enable more predictive outcomes for microbiome engineering (12), agriculture (13), and wastewater systems (6) (Fig. 1c). Here, we expand on this concept by exemplifying the utility of community coalescence pertinent to managed systems and offer insight into how aspects of community ecology should inform the engineering of microbial communities.

**FIG 2:**
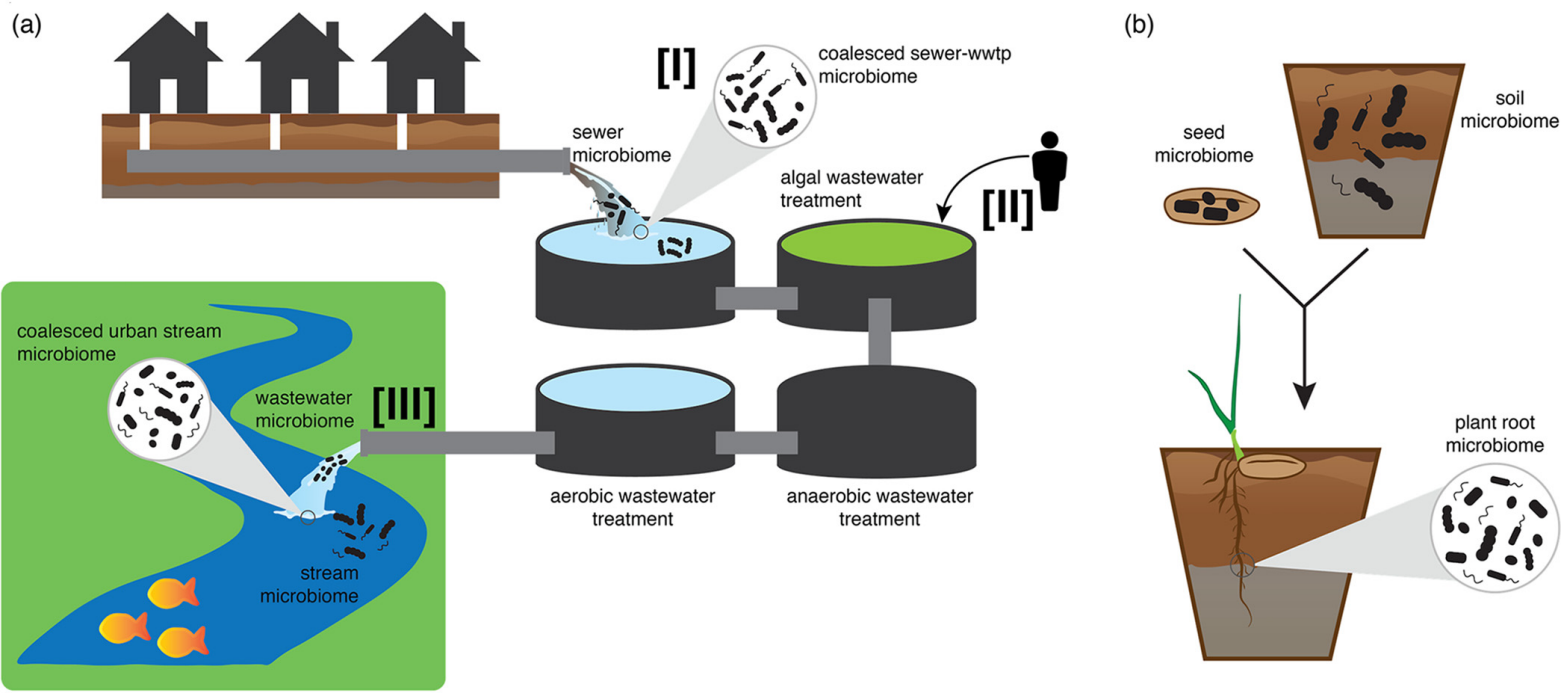
Examples of community coalescences in managed systems in (a) wastewater and (b) seed-soil systems. We use the wastewater system (WWTP) in panel a to illustrate various forms of managed coalescence: (I) redirected coalescence, where municipal effluent occurs regardless, but allowing raw sewage to coalesce directly with urban and natural areas is not optimal (41, 42), so WWTPs redirect the coalescence of raw sewage, itself a mixture from urban infrastructure, for preprocessing; (II) intentional application of microbial consortia, which constitutes an engineered microbiome able to withstand repeated coalescence exposure while maintaining desired community function; and (III) mitigating the release of microbiomes back into nature. In panel b, we illustrate coalescence in a host-associated context with the distinct microbiome of a plant seed interacting with the resident soil microbial community to result in the plant root (i.e., rhizosphere) microbiome, where the importance of rare taxon emergence and pathogen inhibition are optimal criteria for engineered seed microbiomes.

We start by introducing one of the most important managed systems—a municipal effluent and wastewater treatment plant (WWTP), where coalescing of communities occurs consistently and smoothly through incidental and intentional merging (Fig. 2a). Entry into the WWTP unidirectionally coalesces eutrophic sewage into a distinct system with acute abiotic disturbances and optimized microbial consortia to reduce the nutrient, microbial, and toxin loads of the effluent (14–16). In other environmental contexts, similar convergence of drastically different microbial communities also occurs with the bidirectional movement of marine waters into freshwater wetlands along every coastal margin, or where leaky mine tailings unidirectionally flow into stream networks. The merged WWTP effluent continues through a series of engineered abiotic disturbances (Fig. 2a) (17), where supplemental microbial consortia are added to optimize nitrification and denitrification in order to mitigate the nutrient load (18). The supplemented microbial consortia in the WWTP are effectively repeated community coalescences, recycled to amend municipal effluent. Like a sourdough bread starter, the consortia are added to the system to acquire a desired function, then subset and retained to repeat the process. As demonstrated *in silico* by Chang et al. (11), imposing repeated community coalescence perturbations on microbial communities can be an effective strategy of artificial community selection for developing high-performing microbial consortia.

Ideally, the reintroduction of the now cleaned WWTP water into the natural environment comprises oligotrophic water and a low-diversity microbial community (Fig. 2a) (18). In other instances, the managed microbiome may instead be optimized for high diversity and to withstand reassembly into the environment. For example, microbial consortia added to agricultural systems for enhanced productivity or reduced pathogen load are intended to persevere in the merged community (19). Reentry of the clean WWTP effluent into natural systems should, however, not persist. Natural coalescences, such as saltwater intrusion, show that the community with higher initial diversity does not necessarily prevail (20–22). Consequently, clean effluent with a very low-diversity microbiome does not unquestionably indicate a successful cleanup and reentry; a comprehensive understanding of the particular system is crucial for predicting what communities will prevail or subside once coalesced with other communities. These pioneer studies indicate that microbial coalescences are often characterized by “surprising” outcomes where dominant taxa are replaced by initially rare ones or where a community of high biomass and diversity is readily replaced by a low-biomass-diversity counterpart. Such observations indicate that coalescences that are extremely pervasive in natural or managed systems present opportunities to understand the predictability of the system or even intervention points to manage microbial communities for a given outcome.

## LEVERAGING COALESCENCES FOR EFFICIENT MICROBIOME ENGINEERING

The previous examples demonstrate that microbial community coalescence represents a massive biotic perturbation capable of modifying all the attributes of microbiomes, including diversity, composition, function, or resilience to disturbances. As we improve our capacity to predict the outcomes of community coalescence in various environmental contexts, it becomes more evident that this framework offers tremendous possibilities to engineer microbial communities to energize or produce targeted functions.

Community coalescence can be harnessed to construct a target microbial community, comprising (i) robust inoculants with a high degree of compositional and functional resistance/resilience to changing conditions (e.g., wastewater treatment plant, agricultural soils) or intentionally not persistent, as would be the ideal target for the WWTP clean outflow, (ii) communities occupying a specific niche or with competitive capacities to prevent (e.g., pests or pathogens) or stimulate (e.g., beneficial or keystone organisms) the presence of specific taxa, (iii) the optimization of microbial consortia performing specific microbial functions (e.g., biogas production from organic waste, ruminal fermentation in animals), and (iv) the restoration of communities following important biotic or abiotic disturbances (e.g., soil remediation, antibiotic treatment, disease).

Performing entire microbial community mixing with or without their source environment may prove more efficient than using the inoculation of a single microbial strain (10). Single-strain inoculation has faced many challenges hampering its success in the past decade, mainly due to a lack of persistence in the target environment leading to a low efficacy and reapplication costs (23, 24). Several examples of successful microbiome engineering, based on community coalescence, offer some degree of assurance in the reliability of the approach. In the human health context, the use of fecal microbiota transplantation to treat several conditions (e.g., Clostridium difficile infection, ulcerative colitis, antibiotic-treated patients) demonstrates the efficiency of the approach to restore microbiomes by outcompeting unwanted taxa (25–27). Other examples with good reliability include soil restoration by mixing complex soil inocula or amendments, highlighting the efficacy of community blending to increase soil fertility via boosted microbial activity, increased biodiversity, and suppressed soilborne pathogens (Fig. 2b) (28–31).

The integration of ecological theory and engineering design principles into microbiome engineering remains challenging, given the complex interplay between environmental factors and biotic interactions (32). Metacommunity theory has proposed mechanisms linking the spatial dynamics of local and regional species pools, particularly the performance or fitness of local and immigrant species in the receiving environments (33). The community coalescence framework builds on earlier models of immigration dynamics by considering a distinct set of assembly processes that involves fusing whole communities along with the respective environments (1). Although this framework was developed to provide a mechanistic understanding of community assembly processes in natural systems, recent work highlights its utility to the ecological principles of optimizing community coalescence in engineering-based management (4, 34). For example, the harnessing of environmental filtering (environments select certain species) and priority effects (early colonizer impacts on the success of subsequent arrivals) (35) can inform the design and construction of target microbial consortia. Preconditioned microbial assemblages may be able to colonize newly blended communities successfully and rapidly to outperform species with undesired functions. This scenario is particularly useful for the maintenance and optimal performance of a target microbiome highly optimized for removing contaminants in wastewater treatment systems during the short water-retention period. Microbial responses to a coalescence event can be modulated by the history and environmental setting of the community (36). Preexposure of populations to a defined and coalesced environment enhances their resistance and recovery capacities when they are confronted with subsequent perturbations (37). In an environment or managed system exposed to perturbations (the change in the environment that resulted in coalescence), species with preexposure are likely to spread genes involved in the tolerance of those perturbations between organisms via horizontal gene transfer and thereby create genetic “memory” in the newly assembled communities. It is therefore important to consider the history of a source community when designing robust consortia for use in microbiome engineering. Communities subjected to repeated coalescence events may possess desired functions through the repeated biotic disturbance but be highly resistant to subsequent invasions as a potential “side effect” (11). In fact, Chang et al. (11) found that repeated perturbations of coalescence render the microbial communities more resilient to subsequent invasions. This emergence of resilience and resistance may or may not be a desired trait of the engineered consortia. In this regard, understanding how consortia were previously selected and how they respond to subsequent community merging events is crucial information for strategies of titrating the optimal resistance or resilience of the engineered community.

While great advances have been made in understanding microbial community coalescence and applying the theoretical framework in both natural and engineered contexts, many hurdles remain, and it is still difficult to predict outcomes. Community coalescence events often promote new abiotic and biotic conditions where interactions between species with unknown traits occur. Moreover, some degree of stochasticity in community assembly, including priority effects and drift (38), makes the reproducibility of community coalescence difficult to predict in engineered systems. Likewise, the roles of higher-order interactions (32, 39, 40) and trophic interactions (e.g., viral predation) are still largely unknown and therefore make predicting microbiome dynamics challenging. How do we parameterize the collective interactions and account for stochastic events in engineered systems that require an intentional outcome? What are the unknown physiological traits that mediate successful establishment postcoalescence? Despite these challenges, the move from single species to whole community in microbiome engineering clearly demonstrates that systems can be engineered to be robust and resilient (10), with plenty of work yet to be done. The advancement of the community coalescence framework will benefit the design of effective microbiomes by addressing these open questions and will also provide novel insight and interpretations of community dynamics and consequences for ecosystem function.

## Supplementary Material

Reviewer comments

## References

[1] Rillig MC, Tsang A, Roy J . 2016. Microbial community coalescence for microbiome engineering. Front Microbiol 7:1967. doi:10.3389/fmicb.2016.01967. 27999571 PMC5138205

[2] Mansour I, Heppell CM, Ryo M, Rillig MC . 2018. Application of the microbial community coalescence concept to riverine networks. Biol Rev Camb Philos Soc 93:1832–1845. doi:10.1111/brv.12422. 29700966

[3] Castledine M, Buckling A, Padfield D . 2019. A shared coevolutionary history does not alter the outcome of coalescence in experimental populations of Pseudomonas fluorescens. J Evol Biol 32:58–65. doi:10.1111/jeb.13394. 30346633 PMC7379710

[4] Rillig MC, Mansour I . 2017. Microbial ecology: community coalescence stirs things up. Curr Biol 27:R1280–R1282. doi:10.1016/j.cub.2017.10.027. 29207271

[5] Zhou S-Y-D, Li H, Giles M, Neilson R, Yang X-R, Su J-Q . 2020. Microbial flow within an air-phyllosphere-soil continuum. Front Microbiol 11:615481. doi:10.3389/fmicb.2020.615481. 33584580 PMC7873851

[6] Sierocinski P, Milferstedt K, Bayer F, Großkopf T, Alston M, Bastkowski S, Swarbreck D, Hobbs PJ, Soyer OS, Hamelin J, Buckling A . 2017. A single community dominates structure and function of a mixture of multiple methanogenic communities. Curr Biol 27:3390–3395.e4. doi:10.1016/j.cub.2017.09.056. 29107553

[7] Havenaar R, Brink BT, Huis In ’t Veld JHJ . 1992. Selection of strains for probiotic use, p 209–224. *In* Fuller R (ed), Probiotics: the scientific basis. Springer, Dordrecht, The Netherlands.

[8] Conway PL . 1996. Selection criteria for probiotic microorganisms. Asia Pac J Clin Nutr 5:10–14. 24394458

[9] Santos MS, Nogueira MA, Hungria M . 2019. Microbial inoculants: reviewing the past, discussing the present and previewing an outstanding future for the use of beneficial bacteria in agriculture. AMB Express 9:205. doi:10.1186/s13568-019-0932-0. 31865554 PMC6925611

[10] Swenson W, Wilson DS, Elias R . 2000. Artificial ecosystem selection. Proc Natl Acad Sci USA 97:9110–9114. doi:10.1073/pnas.150237597. 10890915 PMC16830

[11] Chang C-Y, Vila JCC, Bender M, Li R, Mankowski MC, Bassette M, Borden J, Golfier S, Sanchez PGL, Waymack R, Zhu X, Diaz-Colunga J, Estrela S, Rebolleda-Gomez M, Sanchez A . 2021. Engineering complex communities by directed evolution. Nat Ecol Evol 5:1011–1023. doi:10.1038/s41559-021-01457-5. 33986540 PMC8263491

[12] Callewaert C, Lambert J, Van de Wiele T . 2017. Towards a bacterial treatment for armpit malodour. Exp Dermatol 26:388–391. doi:10.1111/exd.13259. 27892611

[13] Morella NM, Weng FC-H, Joubert PM, Metcalf CJE, Lindow S, Koskella B . 2020. Successive passaging of a plant-associated microbiome reveals robust habitat and host genotype-dependent selection. Proc Natl Acad Sci USA 117:1148–1159. 10.1073/pnas.1908600116. 31806755 PMC6969547

[14] Preisner M, Neverova-Dziopak E, Kowalewski Z . 2021. Mitigation of eutrophication caused by wastewater discharge: a simulation-based approach. Ambio 50:413–424. doi:10.1007/s13280-020-01346-4. 32451969 PMC7782632

[15] Gleisberg D, Kandler J, Ulrich H, Hartz P . 1976. Eutrophication and wastewater purification. Angew Chem Int Ed Engl 15:354–365. doi:10.1002/anie.197603541. 821362

[16] Zhang B, Xu X, Zhu L . 2017. Structure and function of the microbial consortia of activated sludge in typical municipal wastewater treatment plants in winter. Sci Rep 7:17930–17911. doi:10.1038/s41598-017-17743-x. 29263391 PMC5738398

[17] Boehler M, Zwickenpflug B, Hollender J, Ternes T, Joss A, Siegrist H . 2012. Removal of micropollutants in municipal wastewater treatment plants by powder-activated carbon. Water Sci Technol 66:2115–2121. doi:10.2166/wst.2012.353. 22949241

[18] Boelee NC, Temmink H, Janssen M, Buisman CJN, Wijffels RH . 2011. Nitrogen and phosphorus removal from municipal wastewater effluent using microalgal biofilms. Water Res 45:5925–5933. doi:10.1016/j.watres.2011.08.044. 21940029

[19] Swapna G, Divya M, Brahmaprakash GP . 2016. Survival of microbial consortium in granular formulations, degradation and release of microorganisms in soil. Ann Plant Sci 5:1348–1352. doi:10.21746/aps.2016.05.004.

[20] Rocca JD, Simonin M, Wright JP, Washburne A, Bernhardt E . 2019. Rare microbial taxa emerge when communities collide: freshwater and marine microbiome responses to experimental mixing. Ecology 101:e02956. 10.1002/ecy.2956. 31840237

[21] Werba JA, Stucy AL, Peralta AL, McCoy MW . 2020. Effects of diversity and coalescence of species assemblages on ecosystem function at the margins of an environmental shift. PeerJ 8:e8608. doi:10.7717/peerj.8608. 32195044 PMC7067187

[22] Izabel-Shen D . 2021. Understanding response of microbial communities to saltwater intrusion through microcosms. Comput Struct Biotechnol J 19:929–933. doi:10.1016/j.csbj.2021.01.021. 33598106 PMC7851333

[23] Ranadheera RDCS, Baines SK, Adams MC . 2010. Importance of food in probiotic efficacy. Food Res Int 43:1–7. doi:10.1016/j.foodres.2009.09.009.

[24] Sessitsch A, Pfaffenbichler N, Mitter B . 2019. Microbiome applications from lab to field: facing complexity. Trends Plant Sci 24:194–198. doi:10.1016/j.tplants.2018.12.004. 30670324

[25] Brandt LJ, Aroniadis OC, Mellow M, Kanatzar A, Kelly C, Park T, Stollman N, Rohlke F, Surawicz C . 2012. Long-term follow-up of colonoscopic fecal microbiota transplant for recurrent Clostridium difficile infection. Am J Gastroenterol 107:1079–1087. doi:10.1038/ajg.2012.60. 22450732

[26] Taur Y, Coyte K, Schluter J, Robilotti E, Figueroa C, Gjonbalaj M, Littmann ER, Ling L, Miller L, Gyaltshen Y, Fontana E, Morjaria S, Gyurkocza B, Perales M-A, Castro-Malaspina H, Tamari R, Ponce D, Koehne G, Barker J, Jakubowski A, Papadopoulos E, Dahi P, Sauter C, Shaffer B, Young JW, Peled J, Meagher RC, Jenq RR, van den Brink MRM, Giralt SA, Pamer EG, Xavier JB . 2018. Reconstitution of the gut microbiota of antibiotic-treated patients by autologous fecal microbiota transplant. Sci Transl Med 10:eaap9489. doi:10.1126/scitranslmed.aap9489. 30257956 PMC6468978

[27] Ding X, Li Q, Li P, Zhang T, Cui B, Ji G, Lu X, Zhang F . 2019. Long-term safety and efficacy of fecal microbiota transplant in active ulcerative colitis. Drug Saf 42:869–880. doi:10.1007/s40264-019-00809-2. 30972640

[28] Ling N, Zhu C, Xue C, Chen H, Duan Y, Peng C, Guo S, Shen Q . 2016. Insight into how organic amendments can shape the soil microbiome in long-term field experiments as revealed by network analysis. Soil Biol Biochem 99:137–149. doi:10.1016/j.soilbio.2016.05.005.

[29] Calderón K, Spor A, Breuil M-C, Bru D, Bizouard F, Violle C, Barnard RL, Philippot L . 2017. Effectiveness of ecological rescue for altered soil microbial communities and functions. ISME J 11:272–283. doi:10.1038/ismej.2016.86. 27341455 PMC5315478

[30] Wubs ERJ, Melchers PD, Bezemer TM . 2018. Potential for synergy in soil inoculation for nature restoration by mixing inocula from different successional stages. Plant Soil 433:147–156. doi:10.1007/s11104-018-3825-0. 30930494 PMC6405189

[31] Vida C, Vicente A, Cazorla FM . 2020. The role of organic amendments to soil for crop protection: induction of suppression of soilborne pathogens. Ann Appl Biol 176:1–15. doi:10.1111/aab.12555.

[32] Lechón P, Clegg T, Cook J, Smith TP, Pawar S . 2021. The role of competition versus cooperation in microbial community coalescence. bioRxiv 10.1101/2021.04.18.440290. PMC8601617 34748540

[33] Leibold MA, Holyoak M, Mouquet N, Amarasekare P, Chase JM, Hoopes MF, Holt RD, Shurin JB, Law R, Tilman D, Loreau M, Gonzalez A . 2004. The metacommunity concept: a framework for multi-scale community ecology. Ecol Lett 7:601–613. doi:10.1111/j.1461-0248.2004.00608.x.

[34] Bernstein HC . 2019. Reconciling ecological and engineering design principles for building microbiomes. mSystems 4:e00106-19. doi:10.1128/mSystems.00106-19. 31138720 PMC6584870

[35] Fukami T . 2015. Historical contingency in community assembly: integrating niches, species pools, and priority effects. Annu Rev Ecol Evol Syst 46:1–23. doi:10.1146/annurev-ecolsys-110411-160340.

[36] Andrade-Linares DR, Lehmann A, Rillig MC . 2016. Microbial stress priming: a meta-analysis. Environ Microbiol 18:1277–1288. doi:10.1111/1462-2920.13223. 26768991

[37] Székely AJ, Langenheder S . 2017. Dispersal timing and drought history influence the response of bacterioplankton to drying-rewetting stress. ISME J 11:1764–1776. doi:10.1038/ismej.2017.55. 28440801 PMC5520042

[38] Tucker CM, Fukami T . 2014. Environmental variability counteracts priority effects to facilitate species coexistence: evidence from nectar microbes. Proc Biol Sci 281:20132637. doi:10.1098/rspb.2013.2637. 24430846 PMC3906935

[39] Grilli J, Barabás G, Michalska-Smith MJ, Allesina S . 2017. Higher-order interactions stabilize dynamics in competitive network models. Nature 548:210–213. doi:10.1038/nature23273. 28746307

[40] Singh P, Baruah G . 2021. Higher order interactions and species coexistence. Theor Ecol 14:71–83. doi:10.1007/s12080-020-00481-8.

[41] Rashid SS, Liu Y-Q . 2020. Assessing environmental impacts of large centralized wastewater treatment plants with combined or separate sewer systems in dry/wet seasons by using LCA. Environ Sci Pollut Res Int 27:15674–15690. doi:10.1007/s11356-020-08038-2. 32080814 PMC7190677

[42] Carey RO, Migliaccio KW . 2009. Contribution of wastewater treatment plant effluents to nutrient dynamics in aquatic systems: a review. Environ Manage 44:205–217. doi:10.1007/s00267-009-9309-5. 19458999

